# Volumetric Measurement of Root Resorption following Molar Mini-Screw Implant Intrusion Using Cone Beam Computed Tomography

**DOI:** 10.1371/journal.pone.0060962

**Published:** 2013-04-09

**Authors:** Wen Li, Fei Chen, Feng Zhang, Wanghui Ding, Qingsong Ye, Jiejun Shi, Baiping Fu

**Affiliations:** 1 Department of Orthodontics, School of Stomatology affiliated to Medical College, Zhejiang University, Hangzhou City, Zhejiang Province, China; 2 Department of Orthodontics, School of Medicine and Dentistry, James Cook University, Townsville, Queensland, Australia; University of Toronto, Canada

## Abstract

**Objective:**

Molar intrusion by mini-screw implantation can cause different degrees of root resorption. However, most methods (2-D and 3-D) used for evaluating root resorption have focused on the root length without considering 3-D resorption. The purpose of this study was to volumetrically evaluate root resorption using cone beam computed tomography(CBCT) after mini-screw implant intrusion.

**Materials and Methods:**

1. The volumes of 32 teeth were measured using CBCT and laser scanning to verify the accuracy of CBCT. 2. Twelve overerupted molars from adult patients were investigated in this study. After mini-screw implants were inserted into the buccal and palatal alveolar bones, 150 g of force was applied to the mini-screw implants on each side to intrude the molars. CBCT images of all patients were taken immediately prior to intrusion and after intrusion. The volumes of the roots were calculated using the Mimics software program. The differences between the pre-intrusion and post-intrusion root volumes were statistically evaluated with a paired-samples t-test. In addition, the losses of the roots were statistically compared with each other using one-way analysis of variance at the *P*<0.05 level.

**Results:**

No statistically significant volume differences were observed between the physical (laser scanning) and CBCT measurements (*P*>0.05). The overerupted molars were significantly intruded (*P*<0.05), and the average intrusion was 3.30±1.60 mm. The differences between the pre-intrusion and post-intrusion root volumes were statistically significant for all of the roots investigated (*P*<0.05). The roots were sorted by volume loss in descending order as follows: mesiobuccal, palatal, and distobuccal. Statistical significance was achieved among the three roots. The average total resorption for each tooth was 58.39±1.54 mm^3^.

**Conclusion:**

Volume measurement using CBCT was able to effectively evaluate root resorption caused by mini-screw intrusion. The highest volume loss was observed in the mesiobuccal root among the three roots of the investigated first molar teeth.

## Introduction

The overeruption of molars usually results from early loss of antagonistic teeth or malocclusion of posterior scissor-bite [1]. It increases the difficulty of dental restoration and the restoration of normal occlusion. However, molar intrusion is considered rather difficult due to resistance at the furcation and interradicular area [2]. To intrude overerupted molars, traditional methods such as conventional fixed appliances and high-pull headgear are limited by multiple factors such as patients’ compliance, discomfort and anchorage loss [3], [4]. In contrast, therapy for overerupted molars in hyperdivergent patients with scissor-bite using traditional orthodontic treatment is a significant challenge. Recently, orthodontic mini-screw implants have been successfully used for tooth intrusion [5]–[7]. They have many advantages, including multiple placement sites, uncomplicated placement and removal procedures, and minimal expense for patients. Unfortunately, molar intrusion can increase the risk of root resorption. The evaluation of root resorption in nearly all of the human studies that have been conducted has been based on periapical radiography or panoramic images, which are not accurate enough for this application [8], [9]. Until recently, some studies have evaluated probable root resorption using cone-beam computed tomography (CBCT), but the studies focused on the lengths of teeth and not three-dimensional resorption [10], [11]. It is known that an absolute cut through the long axis of the tooth is extremely difficult. This technique also leads to missing apical or midroot craters. Therefore, it is necessary to quantitatively measure the root resorption using CBCT to obtain a 3D perspective. To the best of our knowledge, no study has been performed to evaluate root volume changes after intrusion with mini-screws. Thus, the aim of this study was to quantitatively evaluate root resorption following molar intrusion using volumetric measurement based on CBCT.

## Materials and Methods

### Treatment Design

This investigation comprised two parts.

#### Part 1 Accuracy of dental volume measurement using CBCT

Before extraction, 32 teeth from 8 adult patients were measured using CBCT, and the DICOM data sets of the patients were imported into Mimics software 10.0 and reconstructed. After extraction, each tooth was scanned from all aspects using a 3D laser scanner (AutoScan 3D Dental Scanner; accuracy: 15 µm; full jaw scanning: 55 sec; Hangzhou Shining 3D Tech Co., Ltd., China), and the volumes of each tooth were calculated automatically by the scanner.

#### Part 2 Quantitatively evaluate the molar intrusion and root resorption using CBCT after mini-screw implant intrusion

The study sample consisted of 12 patients (4 male and 8 female) in our department who had undergone orthodontic treatment to intrude overerupted molars. The ages of the subjects ranged from 18 to 32 years, with a mean age of 24.3±1.26 years. There were 7 Class I and 5 Class II molar relationships, 3 cases were hyperdivergent (SN-MP>37°), and 2 cases were hypodivergent (SN-MP<28°). All of the teeth being intruded were unrestored and asymptomatic with no evidence of caries, periodontitis, periapical radiolucency, or root resorption.

Before the study was begun, informed consent was obtained from the patients. The protocol was approved by the Ethical Committee of Zhejiang University.

### Molar Intrusion

**Figure 1:**
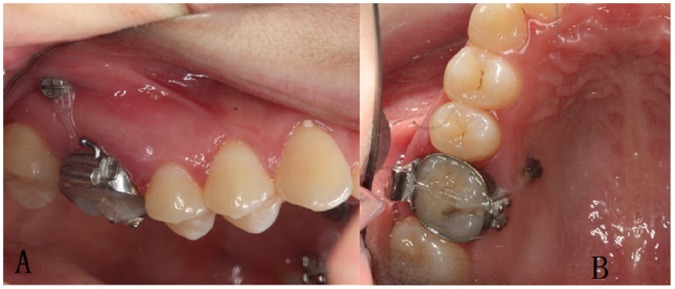
Intra-oral photos of the mini-screw implants. A. The mini-screw implant was placed between the upper first molar and the second molar on the buccal side; B. The mini-screw implant was placed between the first molar and the second premolar on the palatal side.

Pre-treatment and post-intrusion CBCT (NewTom3G QR-DVT9000, QR r.s.I) measurements were taken for each patient. The imaging parameters of the CBCT (field size: large field; detector field: 9 inches; axial thickness: 0.29 mm; tube voltage: 110 kV; maximum current: 15 mA; image element: 0.2–0.4 mm; scan angle: 360°; scan time: 36 s) were set up uniformly. Twelve molars received implantation of both buccal and palatal mini-screw implants (Ti6AL4V(TC4); MB105, Ningbo Cibei Medical Treatment Appliance Co., Ltd., China; diameter: 1.6 mm; implant length: 11 mm; screw length: 7 mm). After local anaesthetic was administered by infiltration, two mini-screw implants were placed between the upper first molar and the second molar on the buccal side and between the first molar and the second premolar on the palatal side (Figure 1).

After placement of mini-screw implants, a medium intrusive force (150 g) was applied immediately using elastic chains. The power chain was activated every 2 weeks. The criteria for sufficient intrusion referred to the American Board of Orthodontics’ Objective Grading System (ABO-OGS): the height difference was less than 0.5 mm between the upper first molar and the second premolar on the adjacent marginal ridge and between the first molar and the second molar on the adjacent marginal ridge [19]. After sufficient intrusion was obtained, the vertical position was maintained by ligating the molars to the mini-screw implants. Nevertheless, particular attention should be paid to oral hygiene.

The average treatment duration for active intrusion was 6±2.6 months with a range of 4 to 9 months.

**Figure 2:**
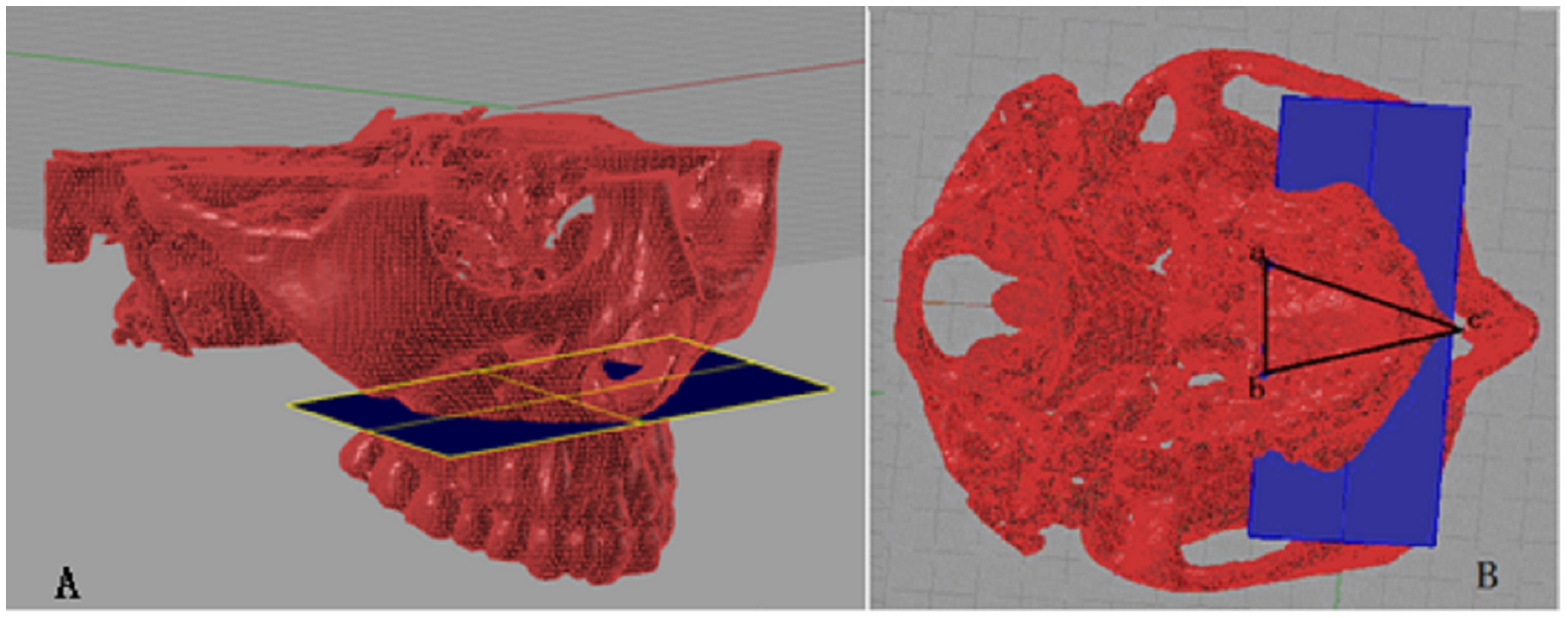
Reference plane for measurement. A. Front view of reference plane; B. Skull base view of reference plane, and three points were identified to ensure the reference plane: a, midpoint of the left greater palatine foramen; b, midpoint of the right greater palatine foramen; c, the forefront of the nasal crest.

**Figure 3:**
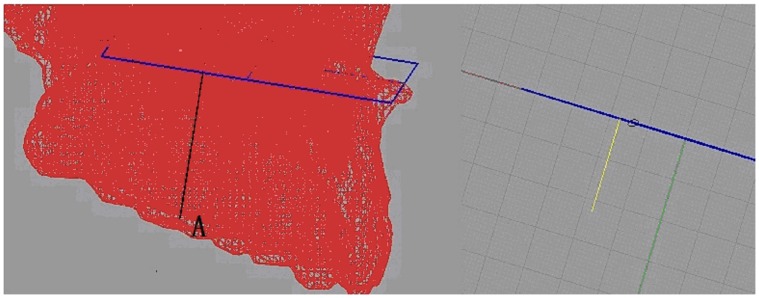
Dental measurements system. A is the crown central fossa (the very low point of fossa).

Tooth intrusion was measured by comparing the pre-intrusion and post-intrusion CBCT radiographs. The DICOM data were imported into the Mimics software 10.0 and reconstructed. To assess dental changes in the vertical direction, landmark images of the crown’s central fossa (the lowest point of the fossa) and reference plane were used to establish a coordinate measurement system. Three points were identified to ensure the reference plane: the midpoint of each greater palatine foramen (maximum diameter of section) and the anterior nasal crest (the forefront of the nasal crest of the sagittal direction) (Figure 2). The distance between the lowest point of the crown’s central fossa and the reference plane was measured (Figure 3). The amount of molar intrusion was represented by the changes in the distance between the mark on the molar occlusional surface and the reference plane.

### Root Resorption

**Figure 4:**
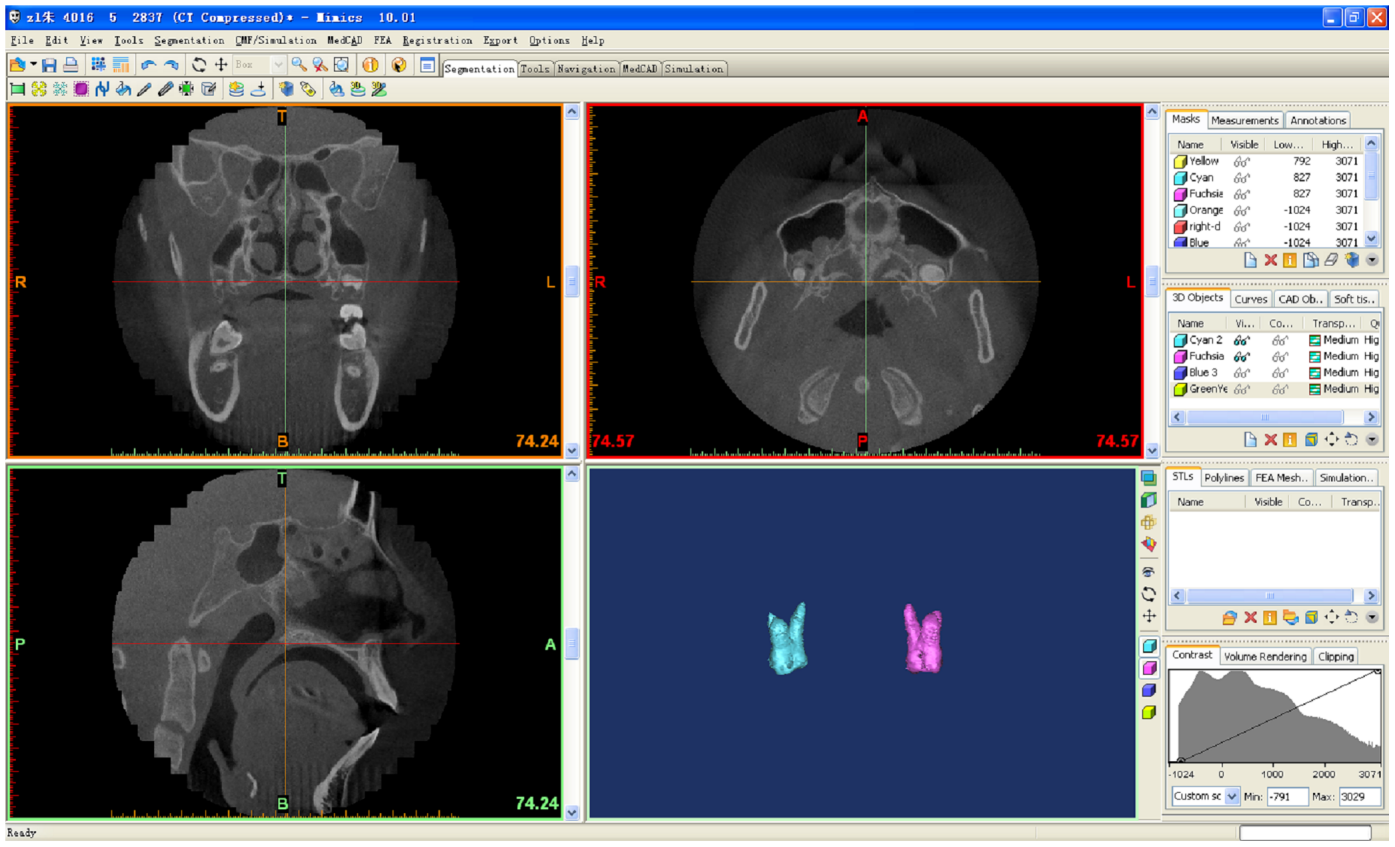
Segmentation of the upper first molar.

**Figure 5:**
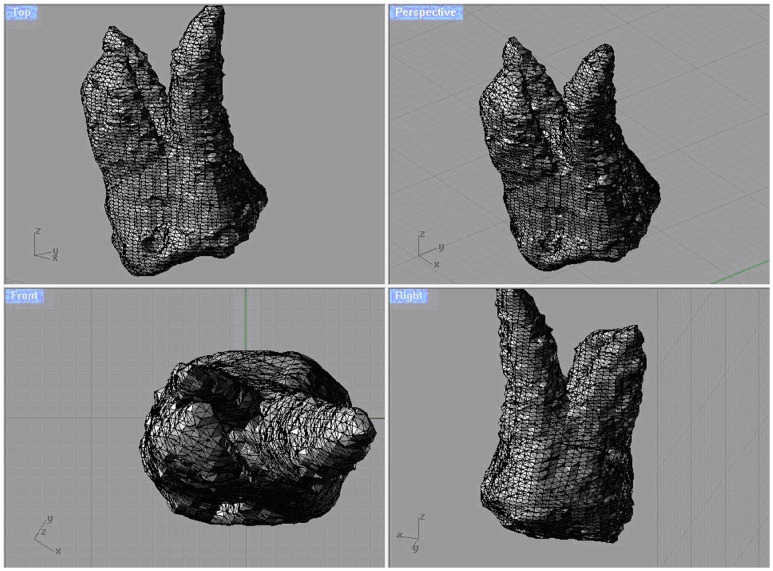
Adjustment of first molar for 3D orientation.

**Figure 6:**
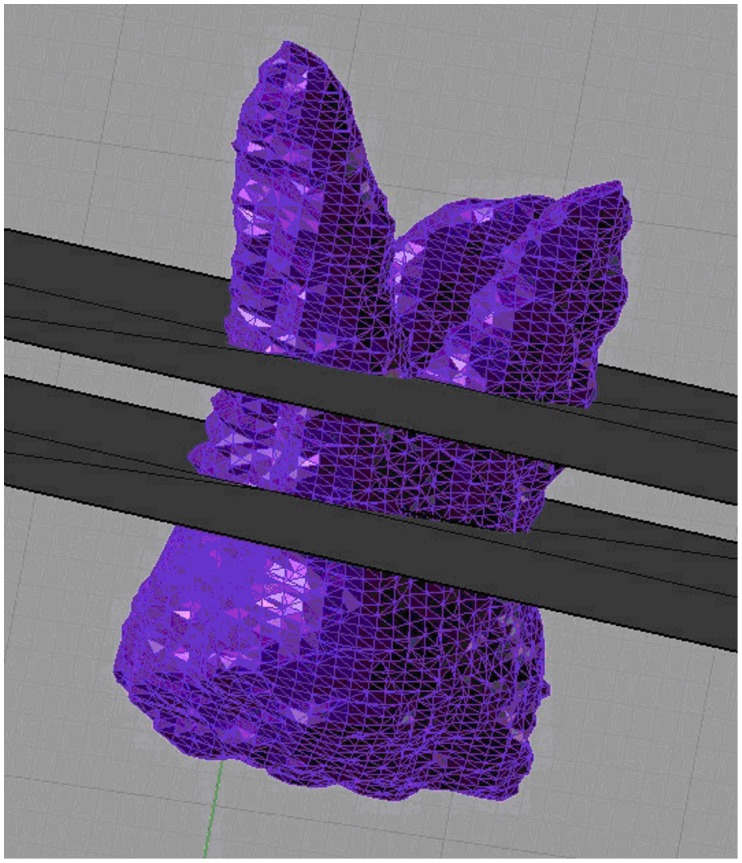
Construction of the reference plane. The plane parallel to the cement-enamel junction and passing through the deepest point of the furcation between these roots.

**Figure 7:**
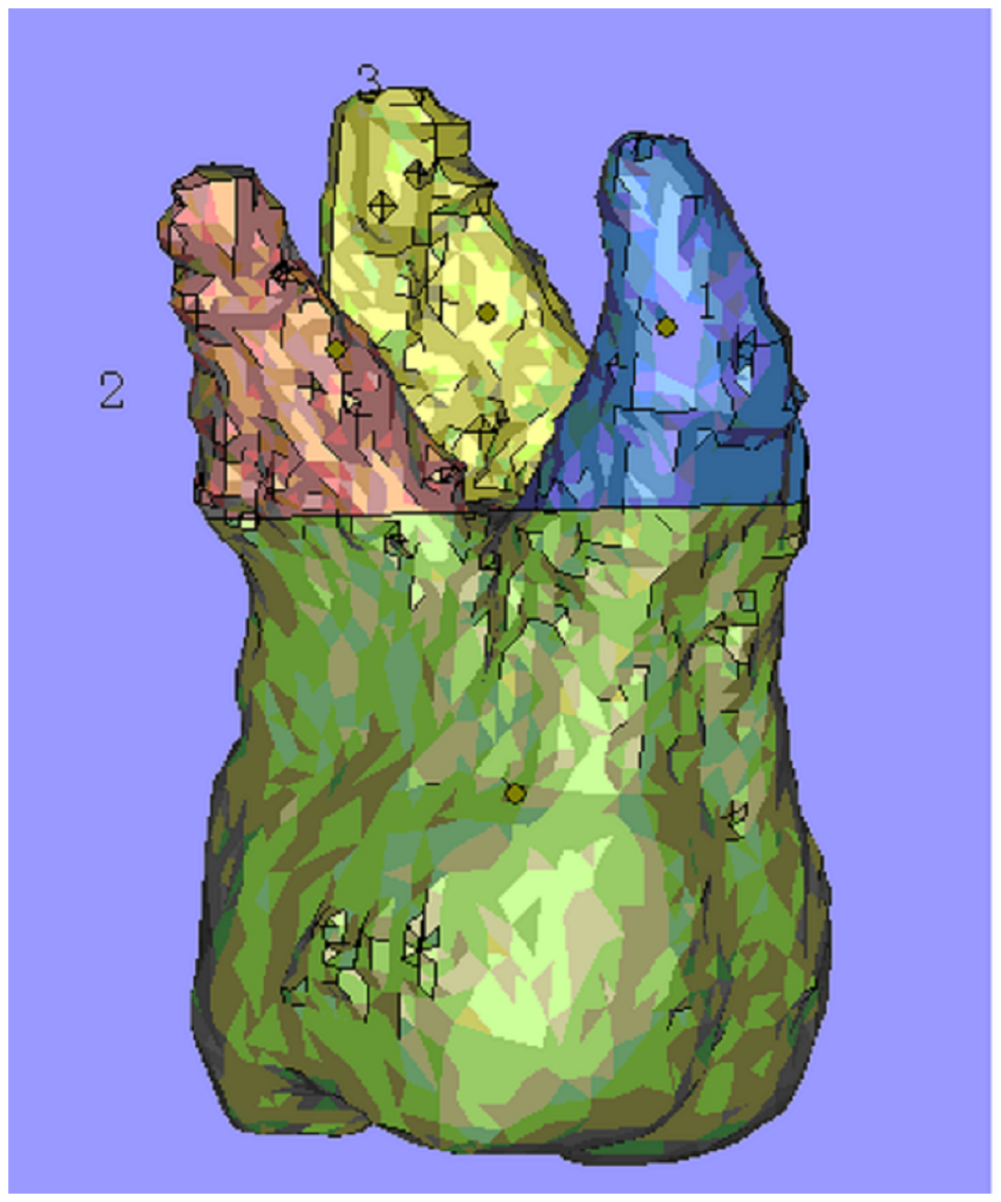
Isolation of the roots of first molar. 1 is the mesiobuccal root; 2 is the distobuccal root; 3 is the palatal root.

Root resorption was evaluated by volumetric measurement [12]. Volume renderings were reconstructed, and the volumetric images were manipulated to display the root surfaces from various orientations. Threshold values were set individually with regard to each patient. The same Hounsfield units (HU) were used for each patient before and after the recordings. On these 3D images, the permanent first molars were conservatively segmented (Figure 4). After segmentation, the permanent first molar teeth were separated from the other teeth and adjusted for 3D orientations (Figure 5). The mesial and distal cusp tips were oriented parallel to the floor. The mesiobuccal and distobuccal roots were separated according to a plane parallel to the cement-enamel junction and passing through the deepest point of the furcation between these roots (Figure 6). Each root was isolated and colour coded. The volume of each root was calculated using the software program (Figure 7). The root volume loss was calculated as the difference between the pre-intrusion and post-intrusion root volumes.

### Method Error

The error of the method was measured to evaluate the reliability of two operators in locating the identification points of the reference plane. Measurements of root volumes as well as the amount of molar intrusion were retaken by two different operators using the same technique. Statistical evaluation of the method error was performed using the paired samples t-test between the initial and repeated measurements.

### Data Analysis

Each data set was tested for normality with the Shapiro-Wilk test. The paired t-test was used to assess the molar intrusion and root resorption before and after treatment. One-way analysis of variance was used to test the percentage of root volume loss. All of the data were expressed as means with standard deviations, and *P*<0.05 was accepted as significant.

## Results

### Method Error

The paired samples t-test revealed that the average difference between any two measurements of molar intrusion was 0.03±0.18 mm, and statistical significance was not achieved. Similarly, the average differences between two measurements of root resorption (mesiobuccal root: −0.23±0.60, *P*>0.05; distobuccal root: −0.06±0.61, *P*>0.05; palatal root: −0.18±0.37, *P*>0.05) were not statistically significant.

### Measurement Accuracy of Dental Volume by CBCT

The dental volume measured using the Mimics software (494.0±70.2 mm^3^) was less than by physical measurement (500.1±68.8 mm^3^). However, the difference between the physical and CBCT measurements was not statistically significant (*P*>0.05).

### Molar Intrusion

None of the mini-screw implants failed during the treatment period. The molars were successfully intruded in all of the patients. Measurements of dental radiographs indicated that the molars were intruded an average of 3.30±1.6 mm over 4 to 9 months (average 6.0±1.59 mon) of force application.

### Root Resorption

Measurements of the root resorption before and after intrusion are given in Table 1. Significant differences were found between the pre-intrusion and post-intrusion measurements for each root volume (*P*<0.05). The highest mean volume loss was recorded for the mesiobuccal root of first molar teeth (22.48±2.58 mm^3^), whereas the distobuccal root showed the least loss (16.22±1.11 mm^3^). Statistical significance was achieved among the three roots (*P*<0.05). The average total resorption for each tooth (58.39±1.54 mm^3^) showed statistical significance between the pre-intrusion and post-intrusion measurements(*P*<0.05).

**Table 1:** Comparison of root resorption after mini-screw intrusion (Mean±SD) (mm^3^).

	n	root resorption amount	*P*
mesiobuccal root	12	22.48±2.58	<0.05^a^
distobuccal root	12	16.22±1.11	<0.05^b^
palatal root	12	19.68±1.49	<0.05^c^

a *P*<0.05,compared to distobuccal root.

b *P*<0.05, compared to palatal root.

c *P*<0.05, compared to mesiobuccal root.

## Discussion

### Molar Intrusion

The present study has indicated that a significant amount of molar intrusion can be achieved with the aid of mini-implants. Though there have been numerous studies on molar intrusion [13],[14], arguments exist in the literature concerning the optimum force to be used for intrusion. These studies have reported force values that varied from 15 to 500 g [15]–[17], but most studies have used forces of 200 g or less [5]. Two reasons may be responsible for this trend. First, excessive strain levels might lead to screw loosening in areas with thin cortical bone and low-density trabecular bone [18]. Second, heavier forces do not increase the rate of intrusion [19]. Some studies have suggested using 40% more than the optimal force when elastic is first applied to compensate for the decrease of force that occurs at the outset [20]. Therefore, the present study began with a force of 150 g [21] knowing that 40% of this force would be lost within the first 12–24 hours. The power chain was activated bimonthly to maintain a constant application of force. Satisfactory intrusion was achieved and the implants showed no loosening. However, further study is needed to provide a biological basis for the optimum force for intrusion.

### Root Resorption

Unfortunately, although the intrusion force is relatively low, root resorption is a common cause for orthodontic treatment. To investigate the incidence and severity of root resorption, many studies have been conducted with the aid of radiography. Previous studies have consistently reported that there is little or no evidence of root resorption associated with the intrusive movement by mini-screw implants; mild root resorption has been observed limited to the cementum at the furcations and the apices, but no root resorption has been observed radiographically [22]. However, these studies were mainly 2-D studies and were not accurate enough to evaluate the amount of resorption. For example, a related study verified that the use of panoramic films to measure pre- and post-treatment root resorption might overestimate the amount of root loss [9].

Recently, the use of CBCT has reversed this situation. CBCT, which provides a lower-dose, higher-accuracy alternative to conventional CT, is being used with increasing frequency in the practice of orthodontics and maxillofacial radiology. Some studies have evaluated probable root resorption using CBCT [10]. They found that external apical root resorption occurred in all of the teeth evaluated, and significant differences were found between the pre- and post-treatment measurements. However, that study only focused on the lengths of the teeth. Because some studies have reported that root resorption has also been found in the mesial, distal and buccal parts of the roots [11], it is necessary to quantitatively detect and measure the root resorption from a 3-D perspective.

We are gratified that three-dimensional volumetric imaging might provide sufficient information to evaluate root resorption [12]. Baysal et al. evaluated the root resorption after rapid maxillary expansion (RME) using CBCT. The results showed that the difference between the pre-expansion and post-expansion root volumes was statistically significant for all of the roots investigated. However, the accuracy of the volume measurements using CBCT has not been evaluated until now. In the first part of our study, the accuracy of the CBCT measurements was tested against a laser scanner whose highest single precision was less than 0.015 mm. No significant difference in volume as measured using the Mimics software and by physical measurement was observed, which indicated that the volume measurement by CBCT was accurate and reliable. In the present study, there was evidence of significant root resorption associated with the intrusive movement as measured using CBCT, which is in disagreement with some previous reports [5], [8], [9], [24]. In our study, all of the investigated roots showed significant root resorption, and the highest mean volume loss was recorded for the mesiobuccal root. The lowest resorption was recorded for the distobuccal root.

The factors relevant to root resorption can be divided into biological and mechanical factors [25]. Mechanical factors include the tooth movement type, force magnitude, duration and type of force and so on. For the biological factors, root morphology, a genetic susceptibility, systemic disease, gender, age and medication intake have been demonstrated to influence root resorption [25]–[27]. In our study, all of the patients denied systemic disease and medication intake. We found that the highest mean volume loss was recorded for the mesiobuccal root of the first molar. Two reasons may be responsible for this finding: the magnitude of the intrusive force and the root form. Some studies have demonstrated that root resorption is related to the magnitude of the force, with resorption increasing as the intrusive forces become stronger [23]. It has also been demonstrated that repair or remodelling of the cementum occurs at the resorbed areas when the force is stopped [28]. These studies suggest that the magnitude of the force is directly related to root resorption. In contrast, atypical root form was a risk factor for root resorption. Some studies have shown that root forms such as eroded, pointed, deviated, and bottle-shaped are prone to resorption [27]. The mesiobuccal root form of the first molar is pointed and a little deviated, which are high-risk factors for root resorption.

Although root resorption was common following molar intrusion, the optimal intrusion force should be found using CBCT measurement, and severe root resorption should be avoided in following studies.

### Conclusion

Volume measurements using CBCT could effectively evaluate the root resorption caused by mini-screw intrusion. The highest volume loss was observed in the mesiobuccal root among the three roots of the investigated first molar teeth.
